# Laying the groundwork: Building relationships for public and patient involvement in pre‐clinical paediatric research

**DOI:** 10.1111/hex.12972

**Published:** 2019-10-18

**Authors:** Wendy Costello, Emma Dorris

**Affiliations:** ^1^ Irish Children's Arthritis Network, Co. Tipperary Ireland; ^2^ UCD Centre for Arthritis Research School of Medicine University College Dublin Dublin Ireland

**Keywords:** biomedical research, communication barriers, communication methods, community participation, laboratory research, paediatrics, patient engagement, public and patient involvement, rheumatology, stakeholder participation

## Abstract

**Context:**

Public and patient involvement is increasingly becoming an expectation of research funders and policy makers. Not all areas of health research are public‐facing. Here, we outline an approach for building the skills and developing the relationships required for downstream public and patient involvement in pre‐clinical adolescent rheumatology research.

**Objective:**

To design a methodology for improving researcher‐adolescent communications specifically aimed at mutual relationship building for PPI. Deliberate and effective preparation in advance of research involvement to improve the downstream success of that involvement.

**Design:**

A research seminar and research skills workshop conducted entirely in ‘plain English’ for adolescents and their siblings aged 10‐20. Upskilling of pre‐clinical researchers for effective public involvement.

**Setting and participants:**

Study co‐design between the voluntary charity Irish Children's Arthritis Network and the academic research centre UCD Centre for Arthritis Research. Fifteen adolescents aged 10‐20 years old living with arthritis, four pre‐clinical researchers and one qualitative researcher investigating adolescent or paediatric arthritis.

**Main variables studied:**

Relationship building and communications for effective downstream public involvement in pre‐clinical and laboratory research.

**Results:**

The methodology outlined here was received extremely positively. Both researchers and adolescents living with arthritis felt more comfortable communicating, more knowledgeable about juvenile arthritis and research, and more able to engage in co‐operative dialogue.

**Discussion:**

Engaging early, considering the needs of the community and developing appropriate involvement methodology can enable involvement in pre‐clinical research.

**Conclusions:**

Dedicating resources to building relationships and skills necessary for co‐operative research involvement can overcome some of the barriers to public involvement in pre‐clinical and laboratory‐based research.

## 1 INTRODUCTION

Science communication is a critical skill for public and patient involvement (PPI) in pre‐clinical and laboratory‐based research. Without some grasp of the relevant science, the public cannot be expected to make informed decisions about research.^1^ Without a pre‐existing relationship, a researcher cannot understand a patient partner's information needs and make their research accessible in a format useful for PPI. It has long been argued that public understanding of science promotes economic prosperity, is an investment in the future and is not a luxury to be indulged in if and when resources allow.^2^ Here, we give an example of how we lay the foundations for future PPI in adolescent rheumatology research by hosting a plain English research seminar for adolescents aged 10‐20 living with rheumatic disease and by hosting a workshop introducing the scientific method. By introducing researchers and their research in an informal setting in advance of developing advisory groups and research partnerships, it gives the public the tools to make more informed decisions around involvement in research and aids the researcher in identifying and building their skills necessary to facilitate future involvement.^3^, ^4^

Bottlenecks in biomedical research that prevented the potential impact of research reaching society have long been acknowledged and gave rise to an area of biomedical research designed explicitly to focus on economically viable innovations that are relevant for and useful clinical or societal applications.^5^, ^6^, ^7^, ^8^ We call this type of biomedical research ‘translational research’.^9^, ^10^, ^11^, ^12^ Given that this area of biomedical research is designed at its core to promote usable research, it seems counterintuitive that PPI is still the exception rather than the norm in this area.^13^, ^14^ There are numerous guidelines for public involvement in laboratory‐based research, but very few are evidence‐based.^15^, ^16^, ^17^ Very few biomedical journals require or indeed ask for public involvement statements for manuscript submission, thereby impeding data collection to build an evidence base for PPI.^17^, ^18^ The reasons for the lag in PPI in biomedical research are multifactorial and include researcher believes and fears; public perspectives and the biomedical system itself.^7^, ^19^, ^20^, ^21^, ^22^, ^23^ A recurring theme from researchers is a lack of peer‐to‐peer learning in this space. Even when a researcher may understand the concept of PPI, its implementation—even with the vast array of available ‘toolkits’—can be challenging.^23^, ^24^

The Patient Voice in Arthritis Research (PVAR) is a PPI initiative established in 2017 and built directly through co‐design with people living with arthritis and rheumatic diseases (hereto referred to under the umbrella term of arthritis).^25^ PVAR was designed specifically to reframe our arthritis research from focusing on the disease to focusing on the patient. Although we carry out multidisciplinary research, the majority of our research is in pre‐clinical and applied biomedical research. We have had excellent success in the adult rheumatology space, with active and on‐going PPI in multiple projects, including biomedical and pre‐clinical research.^26^ We have restructured our academic research centre (the UCD Centre for Arthritis Research) such that patient representatives make up 30% of the steering committee with 1/3 of the voting power. We have also integrated patient representatives onto our interview panels for all publically funded research posts.

Having learned from our experience in the adult space, we are developing our PPI in the paediatric and adolescent arthritis research. However, we had only involved parents and carers of children living with arthritis. Adolescents are not small adults and have their own insight and experience distinct from that of the parent.^27^, ^28^ Our aim is to develop a bespoke PPI initiative to engage and learn directly from children and adolescents living with arthritis. Young people advisory groups (YPAGs) are becoming more common; indeed, the International Children's Advisory Network was established in 2015 as an international network for YPAGs to increase their voice and impact on paediatric medicine and research.^29^ There are examples of adolescent involvement in clinical and social care research, but less so in biomedical research.^30^, ^31^, ^32^ From the researcher perspective, we view PPI as a mechanism for mutual and reciprocal learning with the goal of improving our research relevance and enhancing responsible research practices.^33^ Biomedical research is complex, and in order to meaningfully involve adolescents in research, we first need to ensure we can make our research appropriately accessible. From the patient perspective, our goals are simple, advocate, raise awareness and support our children and young people living with arthritis. We want young people to become more knowledgeable about their disease and feel part of the entire decision‐making process. The objective of this study was to design a methodology for improving researcher: adolescent communications specifically aimed at mutual relationship building for PPI.

## 2 METHODS

### 2.1 Public and patient involvement statement

This was a co‐designed approach with patients or caregivers involved at every stage, including concept (n = 3), design (n = 3), format (n = 3), choice of seminar location (n = 3), choice of research topics (n = 2), target age range (n = 2), review of programme (n = 3), review of presentations/researcher mentoring (n = 26), gathering of feedback (n = 3), and writing and reviewing the manuscript (n = 1).

### 2.2 A research seminar for young people living with arthritis

The inaugural World Young Rheumatic Disease Day (WORD Day) provided an excellent opportunity to bring adolescents and researchers together, as the aim of WORD Day is to raise the awareness and knowledge levels among all stakeholders.^34^ The aim of the research seminar and workshop was to highlight the research on‐going in paediatric arthritis, introduce researchers and adolescents living with arthritis to each other, introduce adolescents to the scientific method and empower adolescents living with arthritis to conduct their own citizen science. The layout of the day was co‐designed between the researchers and iCAN and was crucial to keep the attention of our attendees and make it fun and educational.

### 2.3 Format of the research seminar

Members of the Irish Children's Arthritis Network (iCAN) aged 10‐20 were invited to attend, as were their siblings within that age bracket. As recommended by patient partners during the design of the event, parents were not invited to remain for the seminar. The seminar took place in a neutral, informal venue. Fifteen young people attended and were supervised by members of iCAN: a mentor (aged 19) and two parent members.

Informal introductions were held prior to the first seminar. A microscope with stained joint sections demonstrating a joint with and without inflammatory arthritis was provided, and attendees were encouraged to explore the slides with a researcher and ask questions. A booklet introducing the researchers and their motivations to do research was given to all attendees. Five researchers from different backgrounds and different disciplines presented their research.

We used very strategic scheduling of the presentations to introduce the biomedical research in increasing degrees of complexity, each building upon the last. We had examples of cells taken from the knee joints of people living with arthritis that we use routinely in our biomedical research, which we had brightly stained to make them easily visible, and disseminated to the audience during the biomedical presentations to provide tangible context to the presentations. The biomedical research was bookended by research from public‐facing, more approachable research disciplines:

1. We started with a familiar face to the attendees: a hospital‐based physiotherapist who introduced their clinical research into exercise interventions and why they were doing it.
2. Next, we had an applied biomedical scientist who was working on improving drug design using nanotechnology for a very common arthritis drug. This drug and its side‐effects were well‐known to the attendees. This meant they could understand the motivation and need for the research and, therefore, could more easily grasp the concepts and relevance of the research.
3. We built upon the concept of the cell introduced in presentation two to introduce the immune system and the on‐going research into how an unbalanced immune system can cause disease. This presentation demonstrated how understanding our biology more may lead us to better, faster, evidence‐based treatment choices.
4. The penultimate presentation was from what we considered the most complex and least accessible research: genomic analysis of a rare complex autoinflammatory syndrome. However, presentation three had introduced the concept of both rare disease and autoinflammation, which greatly reduced the burden of complexity on presenter four, and allowed this presentation to focus on how the study of our genes can help to inform us about health and disease. This presentation was by a clinician‐scientist familiar to some of the attendees as a doctor, but who were unaware of the research being undertaken by their clinicians.
5. The final presentation was from a qualitative researcher who discussed their research into online programmes that aim to help young people learn to make informed decisions about their health. They also discussed how they meet with a young person advisory panel of 12‐ to 19‐year‐olds that advise them on how to improve and proceed with their research. This set up the research workshop session as the attendees had an example of how they could potentially help research.

### 2.4 Preparing the researchers

#### 2.4.1 Identifying researcher needs for PPI

The PPI Ready: Researcher Planning Canvas^23^ was used by researchers to identify strategic gaps in the researcher skill sets for patient involvement with adolescents. The PPI Ready planning canvas is freely available and designed specifically for biomedical researchers to reflect on the main theoretical challenges for implementing PPI in advance of starting a research project, such that an individual researcher can address their own perceived challenges to PPI. Our researchers identified communication and comfortable communicating with the target audience as barriers to PPI. A lack of experience communicating with preteens and adolescent also left researchers unsure as to the best method to approach, design and budget for meaningful PPI.

#### 2.4.2 Review of presentations by target age group

All researchers invited to speak were offered the opportunity to have their presentation reviewed in advance of the seminar. Two of the biomedical researchers took this opportunity. We worked with a local school to combine scientific outreach, increased awareness of paediatric arthritis, and to improve our researchers' communication skills. Working with a class of 28 students, aged 11‐12, researchers presented their research and asked for feedback. Together with the class, we brainstormed possible improvements and specifically how to improve the research accessibility. This leads to a number of changes in the final presentations, including the reorganization of presentation structure, improved visual aids, increased interactive elements throughout the day and, crucially, improved overall communication and increased researcher confidence (Box 1).

Box 1 1 **A sweet addition to a Plain English presentation of a complex topic**

Presentation two focused on the complex area of nanobiotechnology, which is used to improve how a drug is delivered into the body with the aim of increasing the efficacy and minimize side‐effects associated with available drugs. The goal of this presenter's research is to encapsulate a common anti‐rheumatic drug and with a view to having it implanted in the joint. Applying the drug locally rather than systemically should vastly reduce the amount of the active drug required and significantly reduce the systemic side effects, which are the most common reason for stopping this medication.

In order to explain this complex concept of nano‐encapsulation, sugar and mints were used. Both were poured into the researcher's hand. The granulated sugar, representing the drug in its standard form, trickled through the researcher's hands, illustrating how the drug cannot remain in the joint (its site of action) in its current form. The mints, however, were contained: just like the goal of his research, to contain the medicine within the joint. This very simple visual was an excellent and effective way of contextualizing the research.

### 2.5 Handing over the tools: an interactive workshop into the research method

If you want to teach people a new way of thinking, don't bother trying to teach them. Instead, give them a tool, the use of which will lead to new ways of thinking. (Richard Buckminster Fuller, scientist and inventor)

Following discussions between iCAN and the Centre for Arthritis Research, we felt a hands‐on, interactive introduction to research skills and the scientific method could act to engage and empower the attendees, increase their understanding of research methodology and help build relationships between the attendees and the researchers. Mutual benefit is a core principle to our PPI initiative and a key concept in the relationship building necessary for successful co‐working relationships, including PPI.^35^, ^36^ Thus, sharing our knowledge and expertise in research skills directly with our attendees had the dual benefit of reinforcing this commitment and building the knowledge base that will be necessary for successful downstream PPI.^4^, ^37^

### 2.6 Format of the research workshop

The seminar room was rearranged to a classic cabaret layout, with workshop‐style tables but which is open‐ended to allow attendees face the presentation area without being closed in. Attendees were divided into four groups, each with a researcher assigned to their table. The basic scientific method was printed as a cartoon in the research seminar handouts, so each person had a reference to hand. Attendees were given the option to vocalize, write or draw any contributions they wished to make. The workshop started with an icebreaker session that everyone in the room took part of, initially in their groups and then together as a whole.

The direction the researchers were given was to act as facilitators and aid participation for all members of their group. They were instructed to get the group to work by asking questions of their group rather than simply giving answers. By design, the researchers did not have knowledge of the workshop tasks in advance and worked as part of their group to figure them out. This was specifically done to help direct the relationship away from patient/researcher towards one of a team working together towards a specific goal. A problem‐based learning pedagogical approach was taken. The workshop was broken into five specific tasks: Observation, Data Management, Clarity of Information, Research Approach and Ethics in research.

#### 2.6.1 OBSERVATION

##### 2.6.1.1 Background

Observation is a critical skill underpinning the scientific method. Observational learning encompasses four key aspects: attention, retention, reproduction and motivation.^38^ This task was designed to highlight these aspects and also to highlight the need for verification of information upon which we base hypotheses.

##### 2.6.1.2 Task instructions

You will be shown a picture of a [named animal] for 5 seconds. You will have 30 seconds to write down/draw as many things about the [named animal]. Team with the most CORRECT observations WINS!

##### 2.6.1.3 Rules

You may not speak or otherwise confer with anyone during this task. To do so will lead to automatic disqualification of the team as a whole. You may draw your observations if you prefer.

At the end of 30 seconds, the picture is put back on the screen and the facilitators (researchers) at each table gather the observations together.

##### 2.6.1.4 The twist

The [named animal] is not in fact the [named animal]. It is another animal that happens to look, when observers are primed, like the [named animal].

##### 2.6.1.5 Learning outcome

This demonstrates the importance of verifying the information you have been given and how observer bias and preconceived ideas can cloud our observational abilities.^39^ The groups picked up on some very fine, minute details whilst missing the fact they had been misled on the larger scale. One researcher and one adolescent attendee (in different groups) noted it was not in fact the [named] anima]l. If the group had not been given preconceived notions about what they were going to observe, the wider group are unlikely to have discounted the big picture observations so readily.^40^, ^41^

#### 2.6.2 DATA MANAGEMENT

##### 2.6.2.1 Background

Responsible management of data is critical in research. Good research data management is critical for responsible, reproducible research.^42^, ^43^

##### 2.6.2.2 Task instructions

The facilitator (researcher) at each table thinks of a word and writes it down unobserved. They then say the word into the left adjacent person's ear, such that it cannot be overheard. That person then passed the word (through speech without being overheard) to the left adjacent person and so on until it has been passed through every person at the table. The last person vocalizes the word, and we compare it to the original word (adapted from the game ‘telephone’).

##### 2.6.2.3 Rules

The word can be said only once. It may not be repeated.

##### 2.6.2.4 Learning outcome

This task highlights the importance of well‐notated source data (the original word written down) and demonstrates how easily data can get distorted. It highlights the importance of systematically documenting observations, as data can easily be misrepresented through unintentional human error.

#### 2.6.3 CLARITY OF INFORMATION

##### 2.6.3.1 Background

How many times have you taken a survey and not understood what the question meant? Clarity is critical for reproducible and reliable data collection.^44^, ^45^

##### 2.6.3.2 Task instructions

A picture of a rectangle was presented. One axis was labelled 10.44 and one axis 2.44. Attendees were simply asked: How long is this rectangle?

##### 2.6.3.3 Learning outcome

A simple question can contain ambiguity. Without labelling of the axes as width or length, there was confusion. As a result, a distribution of answers was given to that apparently very simple question. We brainstormed how to make a clearer question: appropriate labelling, adding units of measurement and additional information on the task as what was precisely required from the participant. This resulted in a much clear question that could avoid confusion and errors in the data.

#### 2.6.4 RESEARCH APPROACH

##### 2.6.4.1 Background

This was the largest task of the day. We used the water strider experiment to brainstorm the approach to research on a given task. The goal of the task was not to successfully complete the experiment, but to take a systematic, evidence‐based approach towards it. A physics experiment was purposely chosen as it was not an expertise of any of the attendees, including the researchers. To start, an image of a water strider was shown. Water striders are small insects adapted for life on top of still water that use the principle of surface tension to ‘walk on water’, even though the insect is more dense than the water.

##### 2.6.4.2 Task instructions

Each group was given a glass with water, tissue paper, a straw, a paper clip and a cotton bud. They were challenged to make the paperclip float on the water.

##### 2.6.4.3 Task instructions part 2

To determine how well the attendees had learned from the other tasks of the day, and to emphasize the importance of being able not just to come up with a solution, but to be able to share it with others, the groups that had successfully floated their paperclip (n = 2) were then challenged to teach the groups who had not succeeded (n = 2).

##### 2.6.4.4 Learning outcomes

This task consolidated the previous learnings and demonstrated the benefit of engaging people with different backgrounds and experiences to problem solve together. It emphasized the importance of trouble‐shooting, hypothesis testing and iterative research design. Furthermore, it demonstrated the need to be able to reproduce your results. It highlighted that producing solutions is only one part of research; you then need to be able to effectively share and communicate research in order for that research to be useful.

#### 2.6.5 ETHICS IN RESEARCH

##### 2.6.5.1 Background

Ethics are the norms for conduct that distinguish between acceptable and unacceptable research behaviour.^46^ Ethical standards govern our conduct in research and are important to produce reliable knowledge and avoid error. Ethics are also essential to collaborative work and integrity in research.^47^

##### 2.6.5.2 Task instructions

As per William Trochim's ‘Teachable tidbit’, we used the example of ‘Facebook and emotional contagion’.^48^ We outlined an impactful study from 2014 that illustrated how you could manipulate a Facebook user's timeline to transfer emotional stress to others and that this can lead people to experience the same emotions without their awareness.^49^ This study has been hugely influential in our understanding of how we can be manipulated via our social media interactions.

###### 2.6.5.2.1 Part 1

Discuss why this study is important and what it might tell us.

###### 2.6.5.2.2 Part 2

Having established the merit of the study, we then dug further into the controversy surrounding the study's ethics. Facebook users were not asked whether they wanted to participate in this study, rather consent was presumed under the terms and conditions of use of Facebook.

Discuss whether you think it was right to presume consent. Why might this have cause ethical controversy?

###### 2.6.5.2.3 Part 3

The three concepts of informed consent, information, comprehension and voluntariness, are introduced.

Brainstorm if these have been met. Discuss again if you think it was right to presume consent.

##### 2.6.5.3 Learning outcomes

Research must be conducted responsibly. Research has the potential to cause harm and must be designed to minimize the potential harm as much as possible. As a researcher, you have a moral and social responsibility to the public. You need to be aware of your responsibilities and conduct your research to the highest ethical standards.

### 2.7 EVALUATION

We used a combination of both leading and lagging indicators as our measures of effectiveness (MOE). Lagging indicators included reported satisfaction of the day. Leading indicators included queries from parents and young people about research and further research events. Based on iCAN's previous experience, we did not use a formal questionnaire approach as we have previously found a lack of depth to the response obtained from this age group. Instead, we used MOE from public postings on the iCAN social media channels and direct queries about future events to iCAN and the PVAR.

## 3 RESULTS

Of the 19 registered, 15 young people attended the event (78.9%). Of those diagnosed with arthritis (n = 9), the age range was 10‐15, with a median age of 14. Of the sibling attendees (n = 6), the age range was 10‐17, with a median age of 14. Registration was open to all young people living with any type of rheumatic disease or their siblings. The majority of attendees had a juvenile idiopathic arthritis (JIA) diagnosis (n = 7), of which n = 1 has systemic JIA, n = 2 have psoriatic JIA and n = 1 had enthesitis‐related arthritis/ankylosing spondylitis JIA. The other two attendees had complex regional pain disorder and hypermobility syndrome, respectively. The overall attendee sex ratio was 4:11 male: female.

A fantastic day. I learned so much about handling my condition… especially how my immune system works…. I have a disease, it doesn't have me.

### 3.1 Attendee

The feedback has been overwhelmingly positive from both the participants, parents and researchers. The focus of the day on the young people, rather than their parents or their disease, was extremely well received. The attendees were encouraged to participate in the research seminar by asking their opinions of the research, rather than simply waiting for them to ask questions. They were asked whether they had any ideas about how to make the research better. This, combined with the neutral location, informal introductions and the interactive element in advance of the research seminar helped with readjusting the traditional power dynamic to allow a more collegiate atmosphere.

The highlight of the day for me was the interactive workshop, the reason being that the researchers sat in groups with us and we became a team together.

### 3.2 Adolescent attendee

The invited age range was 10‐20 years old. The younger side for this spectrum was self‐reported as less likely to grasp the concept of ethics and consent; although they were able to partake in the interactive discussion on a surface level, the more conceptual aspect (such as inherent biases and informed consent) tended to be beyond their interest level. In the future, we would revise our methods to make them more accessible and interesting to this age group. The use of many social media sites, including Facebook, is restricted to those 13 or older due to the Children's Online Privacy Protection Act (COPPA).^50^ Although 38% of 9‐ to 12‐year‐olds have an online social networking presence, this rises to 77% in 13‐ to 16‐year‐olds.^51^ Thus, the reduced familiarity in the younger cohort with social network sites and our use of a task based around Facebook are likely to have contributed to the younger group engaging less in this topic. However, some of the young people (typically those aged 13+ and familiar with social media platforms such as Facebook) participated in avid debate on the topic, demonstrating active engagement with the subject. Similarly, the seminar sessions in the earlier part of the day had mixed reaction depending on age. In particular, the researchers that had never presented to the target audience and who had not availed of the school review (and therefore tended to be less interactive) were the less enjoyable based on audience response. Some of the audiences also felt there may have been too many presentations together and found their focus waning.

Some of it I didn't get, the slides were a bit boring. I found the experiments afterwards very interesting and fun.

### 3.3 Attendee (aged 10)

Alternative, more creative approaches to this topic may improve engagement in the younger age range. The more creative interactive presentations were by far the more preferred style and also the presentations that the young people felt they gained most from. The interactive workshop in the afternoon received the most positive feedback overall.

This is more daunting than speaking to a thousand scientists at a conference.

### 3.4 Laboratory‐based researcher

Preparing the researchers was crucial to the success of the day. Explaining research that is not obvious or accessible to the public is difficult. Good communication needs more than just facts.^52^ Preparing the researchers and having their presentations reviewed and adjusted in response to feedback from the target age group greatly increased their confidence. This not only made their presentations better, but their interactions with the attendees as a whole. This was somewhat time‐consuming for the organizers as it also required wider school outreach and greater context to be included as the target audience was unfamiliar with childhood arthritis. However, both the organizers and the researchers perceived it to be an extremely valuable endeavour that resulted in direct and indirect value to the researchers and to improvement in research communications for the seminar. The feedback from the audience was most positive for the researchers who had availed of this.

As a sibling I found it very interesting to see the research going on into my sister's illness.

### 3.5 Sibling attendee

In order to build the trust required for public involvement, researchers need to find the common ground between themselves and their target partners, generate excitement in their research goals and share information needed for a decision‐making.^53^ The research seminar shared the on‐going research and the researcher's motivations behind it, thereby demonstrating their commitment to the shared goal of improving the lives of those living with arthritis.

## 4 DISCUSSION

[Young person] found it a massively beneficial experience and would definitely attend more. He found the information was given in a very easy to understand way without being overly sympathetic.

### 4.1 Parent of attendee

The concept of public involvement in research is not new.^2^, ^54^ Based on ethical, legal and political principles of autonomy, participative democracy and self‐determination, there has been a movement towards involvement in health care and health care‐related decisions for decades.^55^ Yet, in the pre‐clinical and biomedical field, public involvement in research remains the exception rather than the norm. Increasingly, there is more focus being placed on research impact and the translation and implementation of new scientific evidence in real‐world settings.^37^ Increasing evidence demonstrates that public involvement and stakeholder engagement are key to achieving impact and use of research knowledge.^4^, ^20^, ^56^, ^57^, ^58^

The day was really fun and the researchers interacted with us the whole day. When they explained things it was in a language that we could understand.

### 4.2 Adolescent attendee

Scientific and research literacy can be a barrier to public involvement. Lexicon challenges, mistrust and misconceptions can jeopardize effective communication.^59^ Engaging early, considering the needs of the community with whom you wish to co‐operate and involve, and developing appropriate involvement methodology and public partner roles can all assist in enabling involvement.^15^, ^60^, ^61^ In non‐public‐facing disciplines such as pre‐clinical and biomedical research, significant attention should be given to creating the environment and building the skills required to enable meaningful and successful involvement for all stakeholders.

## 5 FUTURE DIRECTIONS

From a researcher perspective, we firmly believe that understanding the information needs of the public partnerships you engage with is critical for successful PPI.^26^ Often, the unmet needs of the researcher or the need for upskilling in order for a biomedical researcher to feel comfortable enough to meaningfully engage in PPI are overlooked.^23^ Similarly, for unfamiliar research areas or those perceived as complex, more groundwork may need to be done to help young people feel comfortable enough with the topics to fully engage. The methodological approach outlined here was received very positively by both researchers and young people. The interactive nature and learning through doing approach have led to many calls from attendees, parents and young people who did not attend on the day for future interactive, collaborative approaches to opening research to our young people living with arthritis. In direct response, we are in the process of designing future events to make our research more open and accessible to those living with arthritis. The researchers are also keen to continue building relationships and to engage with the adolescents as well as the parents. In the 4 months since the seminar, four rheumatology researchers, including two who were not directly involved on the day, have either initiated or evolved their public involvement to include young people. Furthermore, we have been contacted by biomedical researchers and patient advocates from other disease areas for advice, which was a major catalyst for publishing the approach we used. We are now in the early stage of developing researcher training and evidence‐based approaches to public involvement^62^, ^63^, ^64^, ^65^, ^66^, ^67^ of young people in biomedical research across all disciplines in association with the National Children's Research Centre.

## 6 LIMITATIONS

We acknowledge a number of limitations to this study. The research seminar itself was not accessible to patients with complex medical needs, thereby preventing them from being involved. A recent rapid realist review outlines 33 programme theories to assist with clarifying the mechanisms and resources that enable the reciprocal involvement of seldom heard groups.^68^ These relate to considerations required in the environmental and social planning; service provision; guidelines; financial measures; communication and marketing; and regulation and legislation, many of which may be applicable to improving inclusion in future involvement and events. Furthermore, the seminar was advertised only within iCAN and their networks. This may have excluded interested parties that are not iCAN members. We did not measure baseline attitudes to research and research involvement prior to attendance, thereby preventing a comparative approach to be taken.

## CONFLICT OF INTEREST

The authors have no conflict of interests to declare.

## Data Availability

The data that support the findings of this study are available from the corresponding author upon reasonable request.
